# The term basal plate of the human placenta as a source of functional extravillous trophoblast cells

**DOI:** 10.1186/1477-7827-12-7

**Published:** 2014-01-28

**Authors:** Alexandre U Borbely, Silvana Sandri, Isabella R Fernandes, Karen M Prado, Elaine C Cardoso, Simone Correa-Silva, Renata Albuquerque, Martin Knöfler, Patricia Beltrão-Braga, Ana Campa, Estela Bevilacqua

**Affiliations:** 1 Department of Cell and Developmental Biology, Institute of Biomedical Sciences, University of Sao Paulo, Sao Paulo 05508-000, Brazil; 2 Department of Clinical Chemistry, Faculty of Pharmaceutical Sciences, University of Sao Paulo, Sao Paulo 05508-000, Brazil; 3 Department of Surgery, Veterinary Medicine and Zootechnology School, University of Sao Paulo, Sao Paulo 05508-000, Brazil; 4 Department of Obstetrics and Fetal-Maternal Medicine, Reproductive Biology Unit, Medical University of Vienna, Vienna 1090, Austria; 5 School of Arts, Sciences and Humanities, University of Sao Paulo, Sao Paulo 03828-000, Brazil

**Keywords:** Basal plate, Cell isolation, Extravillous trophoblast cells, Invasiveness, Term placenta

## Abstract

**Background:**

Extravillous trophoblast (EVT) cells are of pivotal importance in human embryo implantation and homeostasis of the maternal fetal interface. Invasion of the endometrium by EVT contributes to placental anchorage, spiral artery remodeling, immunological defense, tolerogenic responses, and several collaborative cross talks involved in establishing and maintaining a successful pregnancy. We report here an improved protocol for the isolation of fully differentiated EVT cells from the basal plate of the human term placenta.

**Methods:**

The basal plate was carefully dissected from the villous tissue and the amniochorion membrane prior to enzymatic digestion. Term basal EVT cells were isolated using a 30 and 60% Percoll gradient. A panel of markers and characteristics of the isolated cells were used to confirm the specificity and efficiency of the method so that their potential as an investigative tool for placental research could be ascertained.

**Results:**

Isolated cells were immunoreactive for cytokeratin-7 (CK-7), placental growth factor, placental alkaline phosphatase, human leukocyte antigen G1 (HLA-G1), and α1 and α5 integrins, similarly to the EVT markers from first trimester placental villi. Around 95% of the isolated cells labeled positively for CK-7 and 82% for HLA-G1. No significant change in viability was observed during 48 h of EVT culture as indicated by propidium iodide incorporation and trypan blue test exclusion. Genes for metalloproteinases MMP-2 and MMP9 (positive regulators of trophoblast invasiveness) were expressed up to 48 h of culturing, as also the gelatinolytic activity of the isolated cells. Transforming growth factor (TGF)-beta, which inhibits proliferation, migration, and invasiveness of first-trimester EVT cells, also reduced invasion of isolated term EVT cells in transwell assays, whereas epidermal growth factor was a positive modulator.

**Conclusions:**

Term basal plate may be a viable source of functional EVT cells that is an alternative to villous explant-derived EVT cells and cell lines. Isolated term EVT cells may be particularly useful in investigation of the role of trophoblast cells in pathological gestations, in which the precise regulation and interactive ability of extravillous trophoblast has been impaired.

## Background

The placenta is a highly specialized essential organ that includes the chorionic villous tree and decidua. The villi are formed by the syncytiotrophoblast (ST) layer in contact with maternal blood, and the cytotrophoblast (VCT) layer, the source of proliferative cells that gives rise to the ST and the extravillous cytotrophoblast (EVT) from the tips of the villi [1]. EVT differentiate in cell subpopulations, characteristically invasive, forming the trophoblastic shell and, interstitial and endovascular cells, respectively invading the decidua and myometrium and uterine vessels [2]. Invasive behavior of these cells is particularly prominent at the first-trimester, although transient, accurately regulated and spatially confined to the endometrium and part of the myometrium [2].

The biological events involved in trophoblast invasion have been compared to those of neoplastic cells [3], making the mechanisms of the former’s invasiveness of great common interest. A high incidence of invasiveness-related changes in trophoblast in adverse pregnancy outcomes, such as placenta accreta, pre-eclampsia [4] and other gestational diseases also highlights the importance of studying EVT cells [5].

First and second trimester human EVT cells constitute a prominent cell population with highly migratory/invasive activities [2]. At term, the cells are partially replaced by fibrinoid and therefore become less numerous. This cell population derives from the small spindle-shaped diploid cells found in the initial gestational trimester [6,7], having a non-proliferative profile, tendency towards polygonal column formation with polyploidy, and reduced invasive activity [8]. These characteristics have been considered disadvantageous for using term basal plate EVT cells. Use of first and second trimester pregnancy tissues, however, depends on ethical considerations and legal regulations, which differ among countries. Hence, term placentas offer an alternative and more readily available source for EVT preparations. Cultured EVT cells also share common phenotypic characteristics in spite of the different protocols described for the isolation first, second and third trimester cells [9-19]. EVT also play key roles in maternal immunoregulation and the molecular regulatory dialogue at the maternal fetal interface. Absence or unregulated production of regulatory factors has been reported in placental-associated gestational pathologies [4,5,8]. In this context, the study of cytotrophoblast cells directly from these microenvironments would allow the analysis of a multitude of impaired signaling pathways, bringing new ways of interpreting the causes and consequences of these pathological conditions.

Villous tissue, villous explant outgrowth, and amniochorion membrane are useful sources from which trophoblast cells can be isolated [9-19]. Explant cultures of anchoring villi and VCT/EVT cells isolated from villous tissue obtained from first-trimester placentas have also been used successfully to yield migratory, temporarily proliferative cytotrophoblast cells for differentiation and invasion studies [9,12]. The contribution of these procedures to elucidating the physiology and paracrine regulation of trophoblast cells is inestimable [20-23]. Third trimester explants and villous digestion have also been used to yield VCT cells [15,16], although they produced fewer viable and invasive VCT/EVT cells than first trimester explants. Terminally differentiation of third trimester VCT cells in vivo is considered an experimental obstacle [11,24].

Term EVT cells can also be isolated in large quantities from the amniochorion membrane [15,17]. These cells tend to merge in vitro, resulting in giant polyploid multinucleated trophoblast cells [17], which are phenotypically and functionally different from in vivo EVT cells in respect of invasive and interactive behavior. The transformation of mononuclear EVT cells into giant multinucleated trophoblast cells and polyploidization seem to be mechanisms associated with villous trophoblast differentiation [24], but not with the multiple particular features of the in vivo extravillous cytotrophoblast inhabiting the decidua. EVTs from decidual basal plate are also in direct contact to maternal cells, which may be important in getting a better understanding of the invasive and tolerance process promoted by these cells.

Thus, we investigated whether the basal plate from term placenta might be a viable source of fully differentiated EVT cells that can be used as a model of trophoblast biology in healthy and pathological conditions.

## Methods

### Reagents

Collagenase type II, insulin, calcium lactate, sodium pyruvate, nucleosides, Trypan blue and fish skin gelatin (Sigma Chemical Co., St. Louis, USA). Antibiotics, 4′,6′-diamino-2-phenylindole dihydrochloride (DAPI), deoxynuclease (DNAse) type I, Dulbecco’s Modified Eagle Medium: Nutrient Mixture F-12 (DMEM/F12), fetal bovine serum (FBS)*,* fibronectin, Iscove’s Modified Dulbecco’s Medium (IMDM), Tryzol® reagent, SuperScript® First Strand kit, and Taq Polymerase (Invitrogen Carlsbad, USA). Matrigel, transwell inserts and filters (Becton Dickinson, Franklin Lakes, USA). Other reagents were from Merck, Darmstadt, Germany) unless otherwise indicated. The specificities and sources of antibodies are listed in Table 1.

**Table 1:** List of antibodies

**Antibody**	**Isotype**	**Dilution**	**Source**
*Mouse monoclonal anti-human CK-7*	IgG1	1:100	Dako Norden A/S (Glostrup, Denmark)
*Mouse monoclonal anti-human HLA-G1*	IgG1	1:100	Exbio (Prague, Czech Republic)
*Mouse monoclonal anti-human Vm*	IgG2a	1:50	Dako Norden A/S (Glostrup, Denmark)
*Rabbit polyclonal anti-hunan PlAP*	IgG1	Ready to use	Invitrogen (Carlsbad, CA, USA)
*Rabbit polyclonal anti-human PlGF*	IgG1	1:100	Abcam (Cambridge, UK)
*Rabbit polyclonal anti-human α1 integrin*	IgG1	1:100	Millipore (Billerica, MA, USA)
*Mouse monoclonal anti-human α5 integrin*	IgG1	1:50	Abcam (Cambridge, UK)
*Mouse monoclonal anti-human desmoplakin I/II*	IgG1	1:200	Abcam (Cambridge, UK)
*Mouse monoclonal anti-human CD68*	IgG1	1:50	Dako Norden A/S (Glostrup, Denmark)
*Rabbit polyclonal anti-human α6 integrin*	IgG1	1:100	Abcam (Cambridge, UK)
*FITC labeled rabbit anti-mouse IgG*	IgG1	1:20	Dako Norden A/S (Glostrup, Denmark)
*TRITC labeled goat anti-rabbit IgG*	IgG1	1:50	Sigma Chemical Co (St. Louis, MO, USA)
*AlexaFluor 488 labeled rabbit anti-mouse IgG*	IgG1	1:1000	Molecular Probes (Eugene, OR, USA)
*AlexaFluor 568 labeled goat anti-rabbit IgG*	IgG1	1:1000	Molecular Probes (Eugene, OR, USA)
*Zenon® Alexa Fluor 488 anti-mouse*	IgG_1_	See supplier’s instruction	Invitrogen (Carlsbad, CA, USA)
*Zenon® R-phycoerythrin anti-mouse*	IgG_1_	See supplier’s instruction	Invitrogen (Carlsbad, CA, USA)

### Tissue collection

Twenty term placentas (37-40 weeks) were obtained from women having elective Caesarean section with healthy babies with no pregnancy complications. Ethical committee approval for this study was granted by the University Hospital and the Institute of Biomedical Sciences from University of Sao Paulo, and informed consent was obtained before surgery. Pools of first trimester placentas (6-12 weeks) were obtained from legal abortions of uncomplicated pregnancies. The use of these tissues was approved by the ethical committee of the Medical University of Vienna.

### Isolation of EVT cells

**Figure 1:**
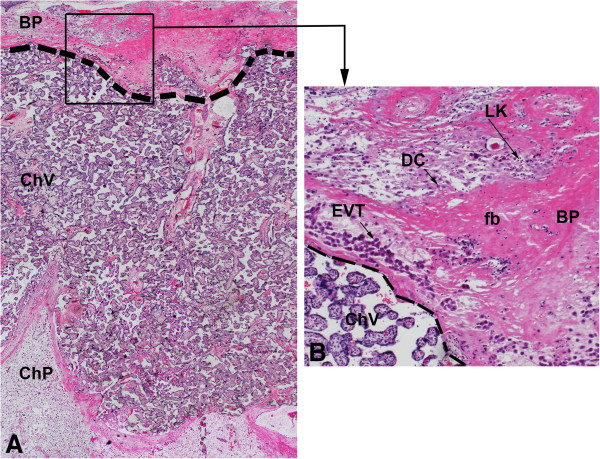
**The term decidual basal plate. (A)** Panoramic view (25x) showing the basal plate (BP), chorionic villi (ChV) and the chorionic plate (ChP) of the human placenta. Dotted line indicates the area initially dissected for isolation of EVT cells. **(B)** High magnification (100x) of the square highlighted in Figure A, in which are indicated extravillous cytotrophoblast cells (EVT), decidual cells (DC) and leukocytes (LK) at the basal plate (BP). (fb) fibrinoid. (ChV) chorionic villi. Hematoxylin-eosin staining.

The term basal plate is a 3-6 mm thick membrane (Figure 1A-B), which was carefully dissected from the villous tissue and the amniochorion membrane. Isolation of EVT cells was adapted from a previously described procedure for amniochorion cytotrophoblast isolation [15,25]. Briefly, the fragments were coarsely minced and ~5 g of wet tissue were incubated for 1 h with 20 mL of DMEM/F12 containing 4% bovine serum albumin (BSA), collagenase type II (125 U/mL) and DNase type I (25 U/mL), at 37°C in a water bath, followed by inactivation with 20% FBS. Cell suspension was double-filtered through a 100 μm mesh followed by a 70 μm mesh. The suspension was centrifuged at 400×g and the cells washed and resuspended in complete DMEM/F12 (supplemented with 1% antibiotics, 10% FBS, 0.01% insulin, 520 μg/mL calcium lactate, 56 μg/mL sodium pyruvate and 1% nucleosides). EVT cells were isolated using a gradient of 30 and 60% Percoll (GE Healthcare, Uppsala, Sweden) followed by centrifugation at 700×g for 30 min [25]. EVT cells were collected from the top of the 30% gradient. When needed, the cell suspension was incubated with 0.83% ammonium chloride in PBS and centrifuged at 400×g for 5 min for blood cell removal.

For some comparative analyses, first trimester EVT were also isolated from pooled villous tissue. The procedure followed steps previously described [26], using adapted enzymatic dispersion and Percoll density gradient centrifugation (GE Healthcare).

### Cell culture

The isolated cells were plated on culture dish plates (Corning Incorporated, Corning, NY, USA) coated with Matrigel (1:1 in DMEM/F12) or fibronectin (10 μg/mL in PBS). Plated cells were incubated for 12h at 37°C under a humidified atmosphere of 5% CO_2_ in air for cell attachment. Non-adherent cells were washed out, and complete DMEM/F12 or IMDM supplemented with 10% FBS was added.

### Immunofluorescence

Isolated cells (3×10^5^ cells/well) plated out for 48 h were fixed in ice-cold methanol. For blocking, 0.05% fish skin gelatin in PBS was used and cells were incubated with primary antibody followed by incubation with fluorescent secondary antibody or incubated with Zenon® Tricolor mouse IgG labeling kit (Table 1). Nuclei were stained with 0.2 μM DAPI. The coverslips were mounted with 1:9 (v/v) PBS/glycerol. Fluorescent cells were quantified as the percentage of reactive cells in the total number of stained nuclei per microscopic field, in a total of 4 fields per sample, with at least 3 samples used on different occasions. Negative controls were developed by adding un-related antibodies.

### Flow cytometry

For cell viability assays, cells (3×10^5^ cells/well) were labeled with 2 μg/mL propidium iodide (PI) and analyzed by FACS Canto® flow cytometry (Becton Dickinson). For intracellular labeling, cells (3×10^5^ cells/well) were incubated in 5% normal goat serum (Vector Laboratories, Burlingame, USA), washed, fixed and permeabilized using commercial buffers (e-Bioscience, San Diego, USA). The cells were incubated in permeabilization buffer with target antibody previously conjugated with a Zenon® Tricolor mouse IgG labeling kit. They were finally resuspended in 0.1% BSA in PBS and analyzed, the data being handled by FlowJo® software (Tree Star Inc., Ashland, USA).

### Trypan blue dye exclusion test

Isolated cells (2.5×10^4^ cells/well) were mixed with 0.4% trypan blue dye in the ratio 1:1 (v/v), and the numbers of viable and dead cells were counted using a hemocytometer.

### RNA extraction and RT–PCR

Total RNA was extracted (3×10^5^ cells/well) using Tryzol. The RNA was assessed by ultraviolet spectrophotometry using a ND-100 spectrophotometer (Nano Drop Technologies, Rockland, USA), and the cDNA was synthesized from 1 μg of total RNA using a SuperScript® First Strand kit. RT–PCR involved Taq polymerase. The primer sequences, annealing temperatures, and PCR product sizes are shown in Table 2. Thermal cycling conditions were 98°C for 2 min, 94°C for 45 sec, 56°C, 60°C, 60°C or 54 for 45 sec, and 72°C for 40 sec, followed by a final extension at 72°C for 5 min. PCR products were visualized with ethidium bromide after electrophoresis on 1% agarose gel. The band densitometric analyses were performed with Image J software (National Institute of Health, Bethesda, Maryland, USA).

**Table 2:** Sequence of primers used in this study

**Gene**	**Sequence**	**Number of cycles**	**Annealing (**°**C)**	**Size**
*18S*	Forward: 5′ GTAACCCGTTGAACCCCATT3′	20	56	115 bp
	Reverse: 5′ CCATCCAATCGGTAGCG 3′			
*GADPH*	Forward: 5′CTGTTGCTGTAGCCAAATTCGT3′	28	60	102 bp
	Reverser: 5′ACCCACTCCTCCACCTTTGA3′			
*MMP-2*	Forward: 5 AGCTCCCGGAAAAGATTGATG3′	35	60	101 bp
	Reverse: 5′ CAGGGTGCTGGCTGAGTAGAT3′			
*MMP-9*	Forward: 5′ GAGGTGGACCGGATGTTCC 3′	35	54	106 bp
	Reverse: 5′ AACTCACGCGCCAGTAGAAG 3′			

### Invasion assay

Invasion was assayed in transwell inserts using 24-well fitted inserts with 8 μm pore size. The inserts were precoated with 15 μL Matrigel (1:1v/v in DMEM/F12) or 30 μL fibronectin (10 μg/mL in PBS). Cells (5.6×10^4^ cells/well) were resuspended in complete DMEM/F12, treated with 10 ng/mL TGF-β or 50 ng/mL EGF, and seeded. The lower chambers were loaded with complete DMEM/F12. Non-invading cells were removed after 48 h, and the membranes fixed and stained with 0.2% violet crystal dye.

### DNA incorporation by BrDUr

Cells plated at 3×10^5^ cells/well and cultured for 24 and 48 h were used to assess proliferation rates. Cultured cells were incubated with 1.5 μg/mL 5-bromo-2′-deoxyuridine (Merck, Darmstadt, Germany) in DMEM/F12 complete medium for 3 h before fixation in 4% paraformaldehyde in PBS for 20 min at room temperature. BrDU was used to detect cells incorporating DNA, using a mouse IgG antibody against BrDU.

### Gelatin zymography

The presence of proteases in the culture supernatants was detected by gelatin zymography. The harvested media were standardized according to the protein content of the cell lysates. Thirty μg total proteins were separated on 10% SDS-PAGE gels containing 5 mg/mL gelatin. The gels were washed in 2.5% Triton X-100 at 37°C and incubated overnight at 37°C in reaction buffer (0.05M Tris-HCl, pH=8.5, 10 mM CaCl2, 1 μM ZnCl_2_). The gels were stained with Coomassie solution (0.5% Coomassie brilliant blue R-250 in 10% methanol and 10% acetic acid) and destained in 10% methanol and 10% acetic acid. Clear zones of gelatin lysis against the blue background stain indicated the presence of gelatinolytic activity. The lysis zones from each sample lane were analyzed using Image J software (National Institute of Health, Bethesda, Maryland, USA).

### Photographic documentation

An Axiovert S100 inverted light microscope (Zeiss, Jena, Germany) was used for general cell morphology and cell counting. Immunofluorescence was analyzed using an Axioskop 2 fluorescence microscope (Zeiss) and the images were taken with the program AxioVision 4.7 (Zeiss).

### Statistical analysis

Statistical analyses were based on one-way analysis of variance followed by a Student-Newman-Keuls multiple comparisons test using Prism v5.0 software from GraphPad (San Diego, CA, USA).

## Results

### Characterization and purity of term basal plate EVT

**Figure 2:**
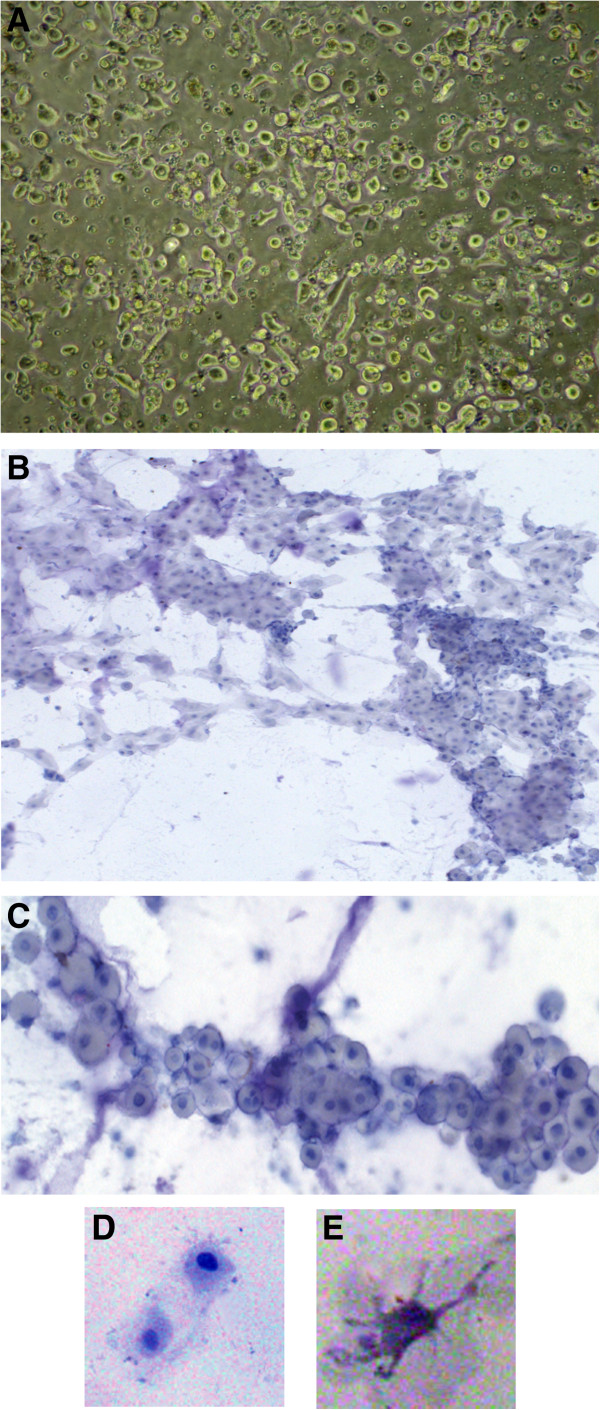
**General morphology of isolated and cultured term basal plate cells. (A)** After isolation (200x). **(B)** Cells organized in cell columns after 24 h of culture on Matrigel (200x). **(C)** Higher magnification of B (400x). **(D-F)** After 48 h of culture, cells exhibit projections indicating migratory activity (1000x). **(A)** Phase-contrast. **(B-F)** Mayer’s hematoxylin staining.

The yield of isolated cells from the basal plate of term placenta was generally around 2-6×10^6^ cells/20 g tissue. After isolation, cells were homogenous, small and roundish in morphology with large nuclei and prominent nucleoli (Figure 2A). The isolated cells were not multinucleated or giant cells. Subsequently, the cells organized into columns (Figure 2B-C) and most showed enlarged polygonal cytoplasm and cell protrusions (Figure 2D-E), suggesting a migratory phenotype.

**Figure 3:**
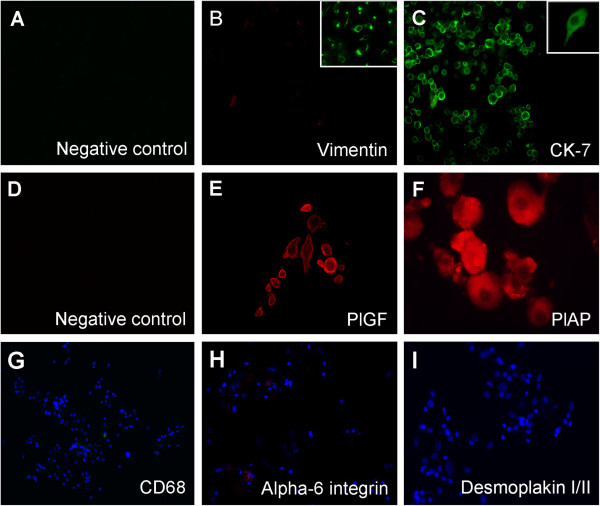
**Representative immunofluorescence characterization and purity of isolated term basal plate cells cultured for 48 h. (A, D)** Negative controls of immunostaining. **(B)** Vimentin staining (200x); insert shows vimentin-expressing endometrial stromal cells (200x). **(C)** CK-7 staining (200x); insert shows a CK-7 positive cell exhibiting projections (1000x). **(E)** PlGF staining (400x). **(F)** PlAP staining (200x). **(G)** CD68 staining (200x). **(H)** Alpha6-integrin staining (200x). **(I)** Desmoplakin I/II staining (200x).

**Figure 4:**
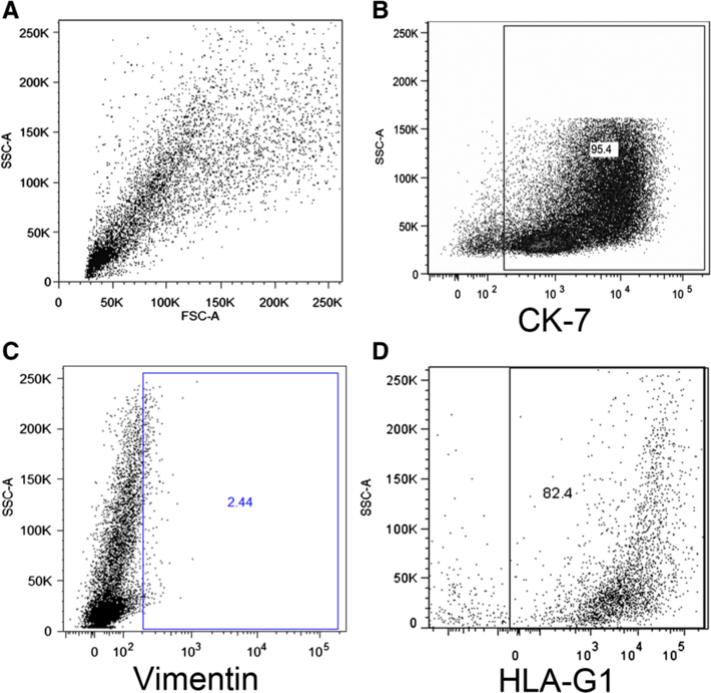
**Representative flow cytometry characterization and purity of isolated term basal plate cells cultured for 48 h.** Flow cytometry plots show cell size and cytoplasmic granularity **(A)**, the amount of CK-7 positive cells **(B)**, the amount of vimentin positive cells **(C)** and HLA-G1 positive cells **(D)**. The data are representative of three independent assays.

The isolated cells have not stained for vimentin (Vm), but reacted positively to cytokeratin (CK)-7 (Figure 3B-C). Cultures of endometrial stromal cells positive to anti-vimentin were used as positive control (Figure 3B – insert). Most were also positive for placental growth factor (PlGF) and placental alkaline phosphatase (PlAP) (Figure 3E-F). Moreover, the great majority of these cells were negative for CD68 (Figure 3G), α6 integrin (Figure 3H) and desmoplakin I/II (Figure 3I). By flow cytometry, an average of 95% of these cells were CK-7 positive, negative for Vm and 82% positive for human leukocyte antigen G1(HLA-G1), corroborating the immunofluorescence findings (Figure 4A-D).

**Figure 5:**
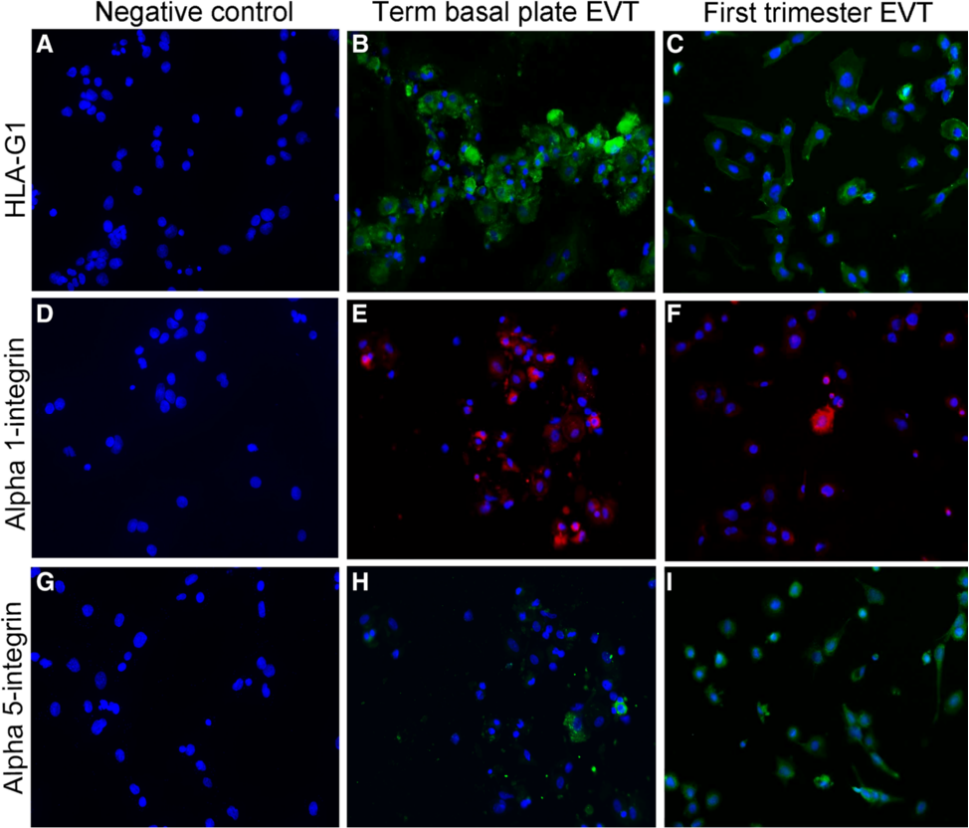
**Term basal plate EVT cells express the main EVT markers similarly to first trimester EVT cells cultured on fibronectin for 48 h. (A,D,G)** Negative controls of the immunofluorescence reactions (200x). **(B-C)** HLA-G staining (200x). **(E-F)** Alpha1-integrin staining (200x). **(H-I)** Alpha5-integrin staining (200x).

First trimester EVT cells contain a specific repertoire of adhesion molecules, closely related to the invasive process, and special antigens from the histocompatibility complex. The persistence of these characteristics in 48 h-cultured term EVT population was also assessed and compared by immunofluorescence for HLA-G1 HLA-G1 (Figure 5B-C), 1 integrin (Figure 5E-F) and 5 integrin (Figure 5H-I). The percentages of HLA-G1 were quite similar between both groups, although first trimester EVT presented higher percentages of both 1 and 5 integrin expression (summarized in Table 3).

**Table 3:** Quantitative analysis of 48 h cultured EVT-markers

	**CK-7 (%)**	**HLA-G (%)**	**α1- integrin (%)**	**α5- integrin (%)**
*Term Basal plate EVT*	95±3	72±7	52±9	53±16
*First trimester villous EVT*	94±5	71±9	64±3	78±7

A total amount of 200 DAP-I positive cells were counted from four randomly microscopic fields on 200x magnification/slide (*n*=3 slides). At the same microscopic fields using appropriated filters, double-positivity cells for CK-7 and, HLA-G1, alpha1-integrin or alpha5 integrin was also counted. The values were expressed as a percentage of the total DAP-I positive cells (According to Pollheimer et al., [26]).

### Cultured term basal plate EVTs are viable and preserve mRNA expression

**Figure 6:**
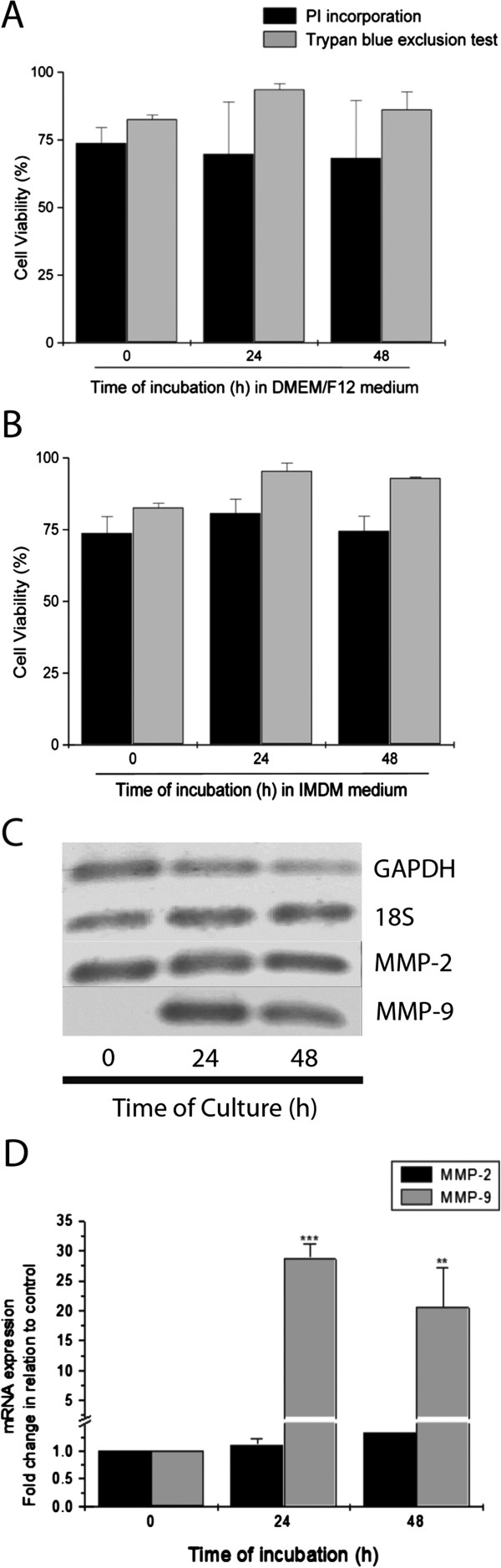
**Cell viability and mRNA expression of term basal plate EVT cells at different times of culture.** Cell viability was analyzed by PI incorporation and trypan blue exclusion using complete DMEM/F12 **(A)** and supplemented IMDM **(B)**. The data represent the mean±SEM of four independent assays. **(C)** Agarose gel electrophoresis of amplified PCR products of GAPDH, 18S, MMP-2 and MMP-9 genes. **(D)** Representation of the RT-PCR data as determined by densitometric analysis of gel bands expressed as a ratio of GAPDH expression. The data represent the means±SEM from three independent experiments. **p<0.01; ***p<0.001 *versus* control.

Cell viability was assessed by PI incorporation and the trypan blue exclusion method at 24 and 48 h of culturing using different culture media with varying composition. Compared to DMEM/F12, IMDM medium has additional amino acids, vitamins and inorganic salts, besides HEPES and selenium, which could be an advantage to improve cell proliferation and viability. The two different types of medium used during cell culturing, however, have no effects on cell viability (Figure 6A-B) and proliferation. Proliferation assessed by BrDU showed that EVT cells were indeed not proliferating, as expected for terminal differentiated EVT cells (data not shown). Gene expression in term EVT cell was preserved during the culture time, as indicated by GADPH and 18S gene expression (Figure 6C).

### Metalloproteinase expression and gelatinolytic activity

**Figure 7:**
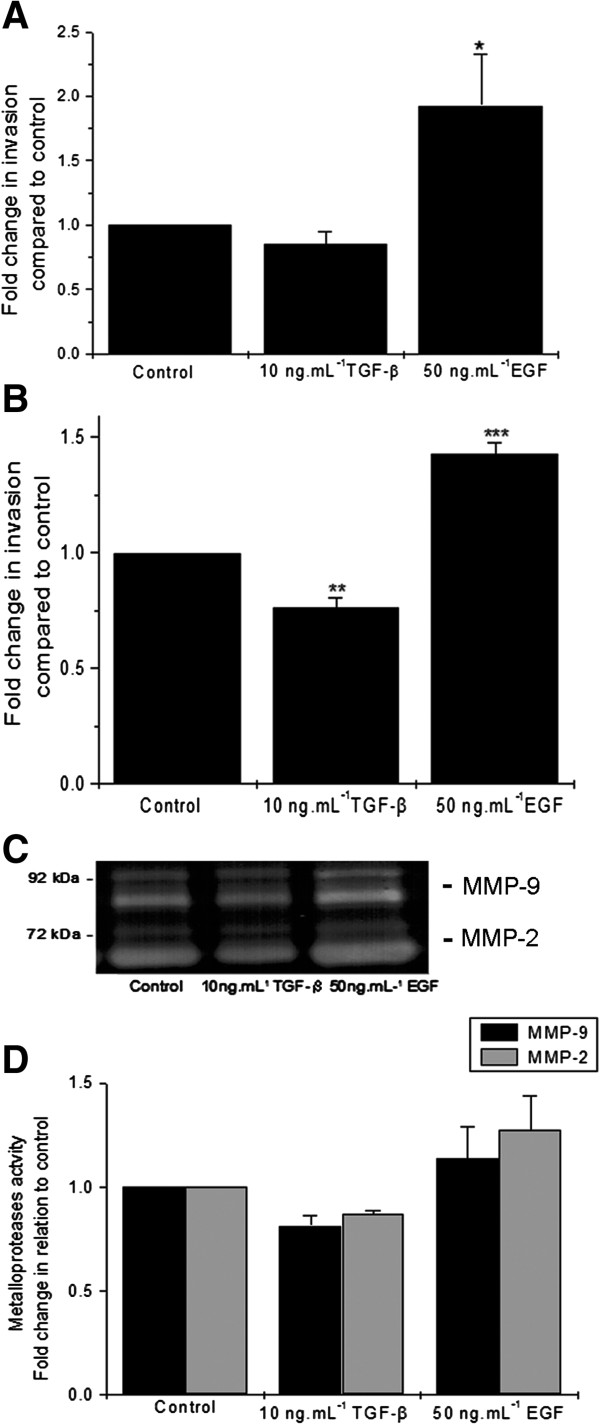
**Term EVT cells maintain their capacity to invade.** TGF-β and EGF modulate the invasion of basal plate EVT maintained in **(A)** Matrigel or **(B)** fibronectin coated transwell inserts and cultured for 48 h. *p<0.05; **p<0.01; ***p<0.001 *versus* control. **(C)** MMP-2 and MMP-9 activities in the presence of TGF-β and EGF in basal plate EVT cultured for 48 h on Matrigel. **(D)** Results of densitometric analysis of gel electrophoresis expressed as fold change in relation to control cultures. In panels **A**, **B** and **D** the data represent the means±SEM of three independent experiments.

MMP-2 and MMP-9 mRNA expression was investigated as key molecules to cell invasion. MMP-2 mRNA was expressed at all times of culture (Figure 6C-D). In contrast, expression of MMP-9 mRNA was low expressed after isolation, but it increased at 24 h and thereafter (Figure 6C-D). Proteolytic activity of cultured cells was also measured in the culture supernatants using gelatin zymography; gelatinolytic activity was seen when EVT cells were cultured on Matrigel (Figure 7C). This activity increased in the presence of EGF and decreased in the presence of TGF-β relative to the control (Figure 7D). The effects of EGF and TGF-β were not so evident in the presence of fibronectin.

### Term basal plate EVT maintains their invasive properties

The invasion potential of EVT cells was evaluated by transwell inserts coated with Matrigel and fibronectin in the presence of TGF-β and EGF at concentration of 10 and 50 ng/mL, respectively. TGF-β decreased invasion, whereas EGF led to a significant enhancement of invasive activity (Figure 7A-B). No changes in proliferation rates were seen after TGF-β and EGF addition to the culture system (data not shown).

## Discussion

We have shown that term basal plate can be a source of viable and functional EVT cells. Isolated EVT cells were positive for CK-7, PlAP, PlGF, HLA-G, and α1 and α5 integrins, the latest three markers also found in first trimester EVT. The viability of these cells and gene transcription along the culture times indicate the suitability of the methods for EVT maintenance. In addition, term EVT cells also respond differentially to regulatory molecules that inhibit or stimulate cell invasion, expressing MMP-2 and MMP-9 as well as showing gelatinolytic activity.

In this study we established a standardized procedure that was successfully applied to the isolation of EVT cells from term placentas. This protocol adapted the enzymatic cell dissociation, by using an enzymatic cocktail instead of trypsin digestion. This procedure resulted in high number of isolated and viable cells, from which EVT cells were subsequently selected by Percoll.

In contrast to villous and amniochorion membrane EVT-derived cells [15,25], EVT cells are exposed to the surrounding microenvironment of extracellular matrix components, which in turn play vital roles in the functions of these cells [26-28]. An important factor for cell viability may therefore be linked to the availability and/or activity of certain proteins of the ECM, whose activity is mediated by cadherins, integrin-matrix ligand among others. The ability of the collagenase in dissociating EVT cells by only cleaving the peptide bonds in the triple helical collagen molecules [29,30], and therefore preserving surface cell molecules and receptors may be a differential crucial factor in our protocol.

Culturing EVT cells in different culture media could provide a myriad of beneficial results. Therefore, the effect of two culture media on the cell viability of these cells was also tested. DMEM/F12 provided acceptable cell viability rates when appropriately supplemented and similar to IMDM, when added of only insulin, SBF and antibiotics. Thus, culturing cells with any of these media makes the cells grow healthy, maintains their cell characteristics and keeps them differentiated and viable, although none induced proliferation.

EVT cells are the only cell type expressing cytokeratin filaments in the term basal plate, when glands and uterine epithelium are no longer present [31,32]. Thus, the expression of CK-7 and the rare or absence of vimentin and CD68 reactive cells respectively identifying mesenchyme and macrophage cell lineages, as observed herein, confirm the trophoblastic nature of the isolated cells and the high degree of purity of the cell cultures.

Several other markers were also investigated in this study and provided further evidence of the purity of the EVT cell population. The isolated EVT cells expressed PlGF, a key placental angiogenic factor [15,16,18], which was consistent with previous studies showing upregulation of this growth factor by term extravillous trophoblast populations [33,34]. These cells were also reactive to the antibody anti-PlAP, a glycosylphosphatidylinositol-trophoblast anchored protein located on the apical membrane of the syncytiotrophoblast and in EVT cells in the term placental bed, notably in the interstitial cytotrophoblast within the maternal decidua [18,35]. The absence of villi cytotrophoblast and syncytiotrophoblast in our cell preparations were also suggested by the absence of desmoplakin I/II staining, peculiar to these trophoblast cell types [36].

Among all trophoblast markers, HLA-G1 is of special functional and immune importance. It is considered a tolerogenic molecule responsible for the reprogramming of local maternal immune response [37]. HLA-G1 is highly expressed by both endovascular and interstitial EVT [38,39] and increases during trophoblast migration towards the spiral arteries [40] - ref-29. Consistent with the others EVT markers and similar to EVT isolated from early placentas, the isolated term basal plate EVT cells were also reactive to HLA-G1.

EVT cells isolated from term basal plate also seem to preserve their invasive phenotype through the positive expression of α1 and α5 integrins, and the negative expression of α6 integrin, as expressed by first trimester EVT cells [41-44]. These extracellular matrix receptors play a pivotal role in stabilizing EVT cell columns by binding to fibronectin and promoting invasiveness upon interaction with collagens and laminin [41-44].

During the first 48 h, cultured EVT cells show a number of characteristics indicating viable and fully active cells, although without proliferative activity. Limited proliferative capacity, commonly found in fully differentiated cells, apparently does not interfere for the short periods with the biological properties of EVT cells. Prolonged EVT cell culturing, as for many other differentiated cells, results in gradually decreasing viability, suggesting low adaptability to the culture conditions or the absence of maintenance specific factors from the maternal-fetal interface [17].

Activation of MMPs is required for first trimester EVT invasion [24]. We have also shown term basal plate EVT cells express MMP-2 and MMP-9 mRNA, and active gelatinolytic secretion, showing that term EVTs have potential to invade. As in other EVT invasive models [21,45], term EVT are also modulated by TGF-β and EGF, in which EGF increases and TGF-β decreases invasion. These data reinforce the term basal plate EVT potential for invasion. Although *in situ* the invasive EVT activity at term is considered down regulated/suppressed, clearly the culture conditions could change this phenotype. As expected, culture conditions did not reproduce the plethora of regulatory molecules, ECM components, hormones and other factors that constitute the placental microenvironment. These data, however, emphasize the plasticity of term EVT cells in response to different stimuli and conditions, making them suitable for experimental purposes.

Different EVT models, regardless of their sources, can provide cells of quality and specificity for a number of experimental analyses. The value of VCT cells derived from first/second trimester villi for invasion studies is irrefutable; these cells develop invasive abilities *in vitro,* although *in vivo* some of them may never interact directly with the endometrial tissue, remaining as floating villi. Term basal plate EVT, on the other hand, beside functionally active in invasion, is fully differentiated, has already invaded maternal tissues, and has been in contact with ECM, maternal immune and non-immune cells throughout gestation. In this regard, these cells are excellent candidate for studies concerning the interrelationship between maternal immune, vascular and decidual systems, and trophoblast. EVT cells isolated from term basal plate also have the advantage of not fusing to form syncytia or multinucleated cells, as seen in amniochorion EVT [8,15].

Although EVT cells are abundant and easily isolated in first trimester placenta, it is not always possible to determine whether they come from a healthy gestation, as the interval between the functional unbalance and the first signs of one disease can be of several weeks. Given that, whether samples from the first trimester were committed to some further gestational pathology remains unknown.

## Conclusions

An adapted method for isolating fully differentiated term basal plate EVT has been presented, which may provide a reliable *in vitro* model for advancing our understanding of a number of processes regulating trophoblast biology. Accordingly, term EVT cells can support future studies on the differences between first and third trimester physiology, and most importantly, comparisons with third trimester pathological gestations, even those associated with EVT-invasion deficiencies. Finally, this would circumvent ethical problems regarding the use of first trimester placenta in countries where abortion is not allowed.

## Competing interests

Authors declare that they have no competing interests.

## Authors’ contributions

AUB standardized the isolation and culture of EVT cells, carried out immunofluorescence and functional assays and drafted the manuscript. SS standardized the isolation and culture of EVT cells, carried out gelatin zymography and PCR assays and drafted the manuscript. IRF helped with isolation and culture of EVT cells. KMP participated on isolation of EVT cells and immunofluorescence assays. ECC, SCS and RA participated on flow cytometry assays. MK participated in study design and donated first trimester samples and antibodies. PBB and AC participated in study design. EB conceived of the study, and participated in its design and coordination and helped to draft the manuscript. All authors have read and approved the final manuscript.
